# A two-phase binning algorithm using *l*-mer frequency on groups of non-overlapping reads

**DOI:** 10.1186/s13015-014-0030-4

**Published:** 2015-01-16

**Authors:** Le Van Vinh, Tran Van Lang, Le Thanh Binh, Tran Van Hoai

**Affiliations:** Faculty of Computer Science and Engineering, HCMC University of Technology, 268 Ly Thuong Kiet, Q10, Ho Chi Minh City, Vietnam; Institute of Applied Mechanics and Informatics, Vietnam Academy of Science and Technology (VAST), 01 Mac Dinh Chi, Q1, Ho Chi Minh City, Vietnam; Faculty of Information Technology, Lac Hong University, 10 Huynh Van Nghe, Bien Hoa, Dong Nai Vietnam; Institute of Biotechnology, Vietnam Academy of Science and Technology (VAST), 18 Hoang Quoc Viet, Cau Giay, Ha Noi Vietnam

**Keywords:** Metagenomics, Binning, Next-generation sequencing, Algorithm, *l*-mers frequency

## Abstract

**Background:**

Metagenomics is the study of genetic materials derived directly from complex microbial samples, instead of from culture. One of the crucial steps in metagenomic analysis, referred to as “binning”, is to separate reads into clusters that represent genomes from closely related organisms. Among the existing binning methods, unsupervised methods base the classification on features extracted from reads, and especially taking advantage in case of the limitation of reference database availability. However, their performance, under various aspects, is still being investigated by recent theoretical and empirical studies. The one addressed in this paper is among those efforts to enhance the accuracy of the classification.

**Results:**

This paper presents an unsupervised algorithm, called BiMeta, for binning of reads from different species in a metagenomic dataset. The algorithm consists of two phases. In the first phase of the algorithm, reads are grouped into groups based on overlap information between the reads. The second phase merges the groups by using an observation on *l*-mer frequency distribution of sets of non-overlapping reads. The experimental results on simulated and real datasets showed that BiMeta outperforms three state-of-the-art binning algorithms for both short and long reads (≥700 *bp*) datasets.

**Conclusions:**

This paper developed a novel and efficient algorithm for binning of metagenomic reads, which does not require any reference database. The software implementing the algorithm and all test datasets mentioned in this paper can be downloaded at http://it.hcmute.edu.vn/bioinfo/bimeta/index.htm.

**Electronic supplementary material:**

The online version of this article (doi:10.1186/s13015-014-0030-4) contains supplementary material, which is available to authorized users.

## Background

As the most diverse forms of life on Earth, microbes directly affect on human lives. Thus, the understanding of microbial communities brings benefits in many fields, e.g., human health, food production, and earth sciences [1]. Initial efforts in studying microbial samples usually use traditional methods which only focus on single species in laboratory culture. However, the methods are limited in use because 99% percent of microbes cannot be cultured in the laboratory [2]. Moreover, because a sample obtained from a microbial community may contain many species which interact with both each other and their habitats, a clone culture cannot represent the true state of affairs in nature [3]. Due to the limitations, the traditional methods are gradually replaced by metagenomics which enables the direct study on genomes from an environmental sample without isolating and culturing single organisms in laboratory.

Sanger sequencing technology is used in some initial metagenomic projects [4,5]. Recently, most projects use next generation sequencing technologies, such as 454 pyrosequencing, Illumina Genome Analyzer, AB SOLiD [6,7]. The new sequencing technologies can produce millions of reads with much faster speed and lower cost. However, the length of sequences generated by these technologies are very different. For example, Illumina read length is from 50 to 300 bp, while Roche 454 System can produce reads with the length of 700 bp [8]. Thus, both of analysis tools for long reads and short reads are necessary for metagenomic projects.

Due to a fact that a metagenomic sample contains reads from various organisms, an important problem needed to be solved in a metagenomics project is to separate reads into groups of closely related organisms. It is referred to as *binning problem*. Binning methods can be roughly classified into three main categories: *supervised, semi-supervised*, and *unsupervised methods*.

Supervised methods require reference databases containing genomes or sequences with known taxonomic origin. They can be further divided into two kinds of methods: *composition-based* and *homology-based* methods. Homology-based algorithms [9,10] usually use an alignment tool (e.g., BLAST) for directly aligning DNA fragments to reference databases, whereas composition-based algorithms extract compositional features (e.g., oligonucleotide frequencies, GC-content [11-13]) from reference genomes and use them for classification. Because the majority of microorganisms on Earth remain undiscovered [14], those methods may be not efficient in practice.

Some methods known as semi-supervised techniques are based on identifying variants of highly conserved genes (e.g., recA, 16S rRNA [15]) to classify reads. However, a drawback of the methods is that some species may contain multiple markers and a maker may be shared by many species [16]. Furthermore, in some species, there is a small ratio of their reads containing 16S rRNA genes. For instance, only 0.4*%*, and 2.7*%* of *Xylella*- and *Flexibacter*-like species reads contain 16S rRNA genes, respectively [17].

To deal with the limited availability of reference databases, some unsupervised methods were proposed to perform the classification using features extracted from reads themselves. LikelyBin [18], a method for binning long reads, was implemented by using a Markov Chain Monte Carlo approach in a *l*-mer feature space. The approach models a collection of reads from multiple genomes as multiple stochastic processes. Not using fixed-order Markov chains as LikelyBin, Scimm [19] used interpolated Markov models, so-called variable-order Markov chains, to cluster reads. MetaCluster 2.0 [20], MetaCluster 3.0 [21] and MCluster [22] are also recent algorithms for classifying long reads. Because of only using a compostional feature, not surprisingly, most of the methods are not suitable for binning of short reads which do not contain enough compositional information.

It is quite obvious that unsupervised metagenomic classification for short reads is a challenging task which attracts various methodologies. Instead of only using a compositional feature, some recent methods focus on classifying of short reads by using other features from data observations or a combination of different features. AbundanceBin [23], and Olga *et al.* [24] are recent binning algorithms for short reads that only rely on abundance levels of genomes. Those methods are able to separate reads which belong to genomes of different abundance levels into different groups, but they cannot classify reads from genomes of similar abundance levels. MetaCluster 4.0 [25] is a hybrid method which separates reads into groups using sequence overlapping of the reads, then the method classifies the groups basing on features extracted from all reads in each group. MetaCluster 5.0 [26] is an extension of MetaCluster 4.0 for dealing with the difference of genome abundance levels in data. TOSS [27] is another hybrid algorithm which classifies reads basing on the classification of *l*-mers. This method groups unique *l*-mers into clusters, and then merges the groups by using an additional property that most of *l*-mer repeats (with a sufficient value of *l*) in a set of metagenomic reads are specific to an individual genome. It is definitely stated in [27] that the algorithm is only suitable for separating reads from genomes with similar abundance levels and sharing large phylogenetic distances.

This paper presents a novel unsupervised algorithm to classify reads from different organisms in a metagenomic dataset, called BiMeta (i.e., A *Bi*nning algorithm for *Meta*genomic reads). As the existing hybrid methods mentioned above, BiMeta firstly performs a preprocessing phase which groups reads basing on sequence overlapping information, then it merges the read groups using features extracted from themselves. A new idea contributed in this study different from the others is a way of extracting compositional features of the read groups. Instead of extracting the features from all reads of each group, we compute *l*-mer frequency distribution of their subgroups which only consists of non-overlapping reads. The idea is motivated by an observation conducted by this study that the *l*-mer frequency distribution of a group of non-overlapping reads are unique to each genome.

The next section presents the details of the observation and the proposed algorithm in which the observation is applied. The experiments results and discussions section demonstrates the strength of BiMeta on both simulated and real metagenomic datasets. The final section is for conclusions.

## Methods

### Notations and terms

This section presents some notations and clarifies terms needed for the statements and analysis of methods utilized in this study.

- Given two DNA reads *r* and *s*. If *r* and *s* are sampled from the same genome, we denote it by *r*⋈*s*.
- Given two genomes *g*_1_,*g*_2_, for example:
  - *g*_1_=“CCTAAGAACGGTT”,
  - *g*_2_=“AAGTGTGCTTTAT”.
  Let’s consider 4 following reads possibly extracted from *g*_1_:
  - $r^{\,g_{1}}_{1}$=“CCTAA”(stating at position 1 in *g*_1_),
  - $r^{\,g_{1}}_{2}$=“AAGAA”(at position 4 in *g*_1_),
  - $r^{\,g_{1}}_{3}$=“AACGG”(at position 7 in *g*_1_),
  - $r^{\,g_{1}}_{4}$=“CGGTT”(at position 9 in *g*_1_),
  and one read from *g*_2_:
  - $r^{g_{2}}_{1}$=“AAGTG”(at position 1 in *g*_2_).
  Considering one strand of the DNA sequences:
  - Because *r*1*g*_1_⋈*r*2*g*_1_ and the two reads share a common region of *g*_1_, we say that $r^{g_{1}}_{1}$*correctly overlaps* (or *overlaps* in short) $r^{g_{1}}_{2}$, denoted by *r*1*g*_1_⊓*r*2*g*_1_.
  - We also say that $r^{g_{1}}_{1}$*does not overlap*$r^{g_{1}}_{3}$, $r^{g_{1}}_{4}$, $r^{g_{2}}_{1}$, denoted by *r*1*g*_1_ ⊓̸ *r*3*g*_1_, *r*1*g*_1_ ⊓̸ *r*4*g*_1_, and *r*1*g*_1_ ⊓̸ *r*1*g*_2_. Although $r^{g_{1}}_{1}$ and $r^{g_{1}}_{3}$ share a substring “AA”on the left end of the first read and the right end of the second one, they are not considered to *overlap* in the scope of this paper because they are extracted from different regions of *g*_1_. Similarity, $r^{g_{1}}_{1}$ and $r^{g_{2}}_{1}$ are said not to overlap each other because they are from different genomes.

### Observation of *l*-mer frequency distributions on groups of non-overlapping reads

The *l*-mer frequency is known as a DNA composition feature of each DNA fragment or genome. The authors in papers [20,28,29] have revealed that the short *l*-mer frequency distributions of long fragments or whole genome sequences are unique to each genome. However, most sequencing technologies used in current metagenomic projects cannot produce long fragments. Thus, it is not efficient to directly apply the feature to metagenomic reads classification.

In this study, instead of observing on long DNA fragments, we analyse *l*-mer frequency distributions on groups of non-overlapping short reads. Each group only consists of reads which are sampled from the same genome. This work considers the difference between *l*-mer frequency distributions of read groups from the same and different species genomes as well.

#### Calculation of *l*-mer frequency

An *l*-mer frequency distribution of a read group is computed as follows. Let *G* be a group containing *n* reads: *G*={*r*_*j*_,*j*=1,…,*n*}, and |*r*_*j*_| be the length of *r*_*j*_. Each read *r*_*j*_ consists of |*r*_*j*_|−*l*+1*l*-mers. So, the total number of *l*-mers in group *G* is $|G|=\sum _{j=1}^{n}(|r_{j}| - l + 1)$.

Because *l*-mers are composed of 4 kinds of nucleotides (Adenine (A), Cytosine (C), Guanine (G), and Thymine (T)), there are at most 4^*l*^ possibilities of *l*-mers. Let ${h^{G}_{i}}, i \in \left [1,\ldots, 4^{l}\right ]$ denote the frequency of *l*-mer *i* in read group *G*. To compute ${h^{G}_{i}}$, a sliding window of length *l* is used to slide along all DNA reads of each group. In practice, because groups may have different number of reads, and lengths of reads may be different, this study uses a normalized frequency which is based on the total number of *l*-mers in each group. It can be calculated as follows.

(1) $$ {f^{G}_{i}} = \frac{{h^{G}_{i}}}{|G|}, i =1,\ldots, 4^{l}  $$

where ${f^{G}_{i}}$ be the normalized frequency of *l*-mer *i* in read group *G*. The feature vector of group *G* will be $\mathbf {f^{G}}=\left [{f^{G}_{1}}, {f^{G}_{2}},.., f^{G}_{4^{l}}\right ]$. (For simplicity, from now, we use *frequency* to refer to *normalized frequency*).

In addition, when considering both strands of DNA sequences within each group, because *l*-mers and their reverse complement *l*-mers (e.g., 4-mers: AAAA/TTTT, GCGC/GCGC, ACCC/GGGT) have the same frequencies, a technique as in [20,28] was used to reduce the size of the vector. If *l* is odd, size of the feature vector will be 4^*l*^/2, and if *l* is even, the size will be (4^*l*^+4^*l*/2^)/2. The studies of Chor *et al.* [29] and Zhou *et al.* [28] present that *l*=4 is the best choice to extract compositional features from DNA sequences. In this study, we also choose *l*=4. Therefore, each feature vector of a read group has a size of 136.

#### Extracted compositional features

In this paper, an experiment is conducted to extract compositional features from groups of non-overlapping reads by using the above method of calculation of *l*-mer frequency. Each group consists of 60 error-free sequencing short reads with length of 150 bp. Therefore, the size of each group (i.e., sum of all read lengths in the group) is approximately 9000 bp. In addition, all reads *r* and *s* in the same group are sampled such that *r*⋈*s* and *r* ⊓̸ *s*. There are totally 150 pairs of read groups used in the experiment. Among them there are 50 pairs from the same species genome, 50 pairs from genomes in the same genus but in different species (the phylogenetic distance of species), and 50 pairs from genomes in the same order but in different families (the phylogenetic distance of family).

The Euclidean distance between feature vectors of groups in each pair are computed (The details are given in Additional file 1). Let *u* and *v* be two different species. We denote by *G*^*u*^ and *G*^*v*^ groups which consist of reads belonging to species *u* and *v*, respectively. In the experiment, we realize that:

- The Euclidean distance between feature vectors $\phantom {\dot {i}\!}\mathbf {f}^{G_{1}}$ and $\phantom {\dot {i}\!}\mathbf {f}^{G_{2}}$, denoted by $\phantom {\dot {i}\!}||\mathbf {f}^{G_{1}} - \mathbf {f}^{G_{2}}||$, is quite small if two read groups *G*_1_ and *G*_2_ are sampled from the same species genome (≈7.7×10^−4^ in average).
- $\phantom {\dot {i}\!}||\mathbf {f}^{G^{u}} - \mathbf {f}^{G^{v}}||$ is larger if the phylogenetic distance between *u* and *v* is larger (≈1.4×10^−3^, and ≈2.1×10^−3^ in average for the phylogenic distances of species and family, respectively).

**Figure 1:**
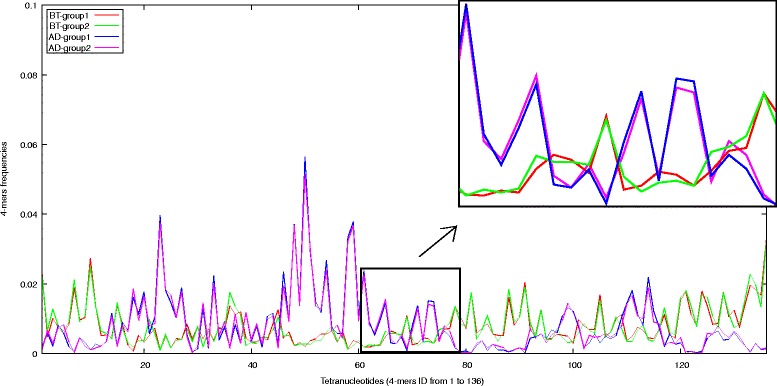
**4-mer frequency distributions of groups of non-overlapping reads.** Four groups sampled from genomes of two species: *Bacillus thuringiensis* (BT-group1, BT-group2) and *Alicycliphilus denitrificans* (AD-group1, AD-group2).

In addition, Figure 1 shows the 4-mer frequency distribution of 4 groups of non-overlapping reads which belong to genomes of two species: *Bacillus thuringiensis* and *Alicycliphilus denitrificans*. Obviously, the read groups are sampled from the same species genome have similar 4-mer frequency distributions, while the 4-mer frequency distributions of the groups from genomes of the different species are very different.

This observation demonstrates that tetranucleotide frequency-based genomic signatures are also preserved in a group of non-overlapping short reads as in long fragments. Thus, it can be used as a feature for organism classification.

### Fundamentals of proposed method

**Figure 2:**
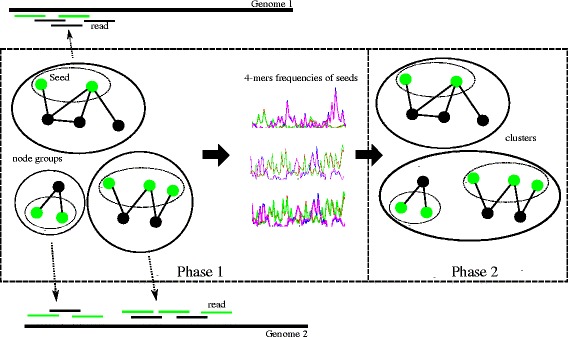
**Binning process of BiMeta.** Phase 1 groups reads into groups, builds and computes 4-mers frequencies of seeds. Phase 2 merges the groups into clusters.

The above observation motivates us to propose a two-phase algorithm for the binning problem of metagenomic reads as follows (Figure 2).

Let *R* be a set of *n* metagenomic reads. In the first phase of the proposed algorithm, the reads are grouped into groups *G*_*i*_,*i*∈{1,…,*p*} and *p*≤*n* basing on their overlapping information. In the other word, two reads *r*,*s*∈*R* can be grouped if it is concluded that *r*⊓*s*. As denoted above, this means that all reads *r*,*s*∈*R* in the same group are regarded as belonging to the same genome (*r*⋈*s*).

In order to merge the groups into clusters which represent genomes from closely related organisms, we compute a feature vector **f** for each group *G*_*i*_. An idea applied in this study is that for each group *G*_*i*_, the proposed method does not need to compute the feature vector **f** on all its reads which can overlap each other. Instead, a subset *S*(*G*_*i*_) of *G*_*i*_, which is concluded to satisfy that ∀*r*,*s*∈*S*(*G*_*i*_),*r* ⊓̸ *s*, is firstly extracted from *G*_*i*_. We call it *a seed* of *G*_*i*_. An example in Figure 2, a group which belongs to Genome 1 consists of 5 reads (presented by 5 lines). A seed of the group consists of 2 reads (presented by 2 green lines) which do not overlap each other in the seed. Next, feature vector $\phantom {\dot {i}\!}\mathbf {f^{\,S(G_{i})}}$ for each subset *S*(*G*_*i*_) is calculated. We expect that ∀*r*,*s*∈*S*(*G*_*i*_),*r*⋈*s* and *r* ⊓̸ *s*, the feature vector $\mathbf {f^{\,S(G_{i})}}\phantom {\dot {i}\!}$ serves as a genomic signature to classify microbial organisms, supported by the observation mentioned above. Thus, $\phantom {\dot {i}\!}\mathbf {f^{\,S(G_{i})}}$ is used as a representative of *G*_*i*_ in the classification process. In the second phase of the proposed algorithm, the read groups *G*_*i*_,*i*∈{1,…,*p*} are merged into *k* clusters (*k*≤*p*) using their feature vectors $\mathbf {f}^{\,S(G_{i})}\phantom {\dot {i}\!}$.

#### Finding overlapping and non-overlapping reads

As mentioned above, a necessary problem which is solved in the first phase of the proposed algorithm is to determine whether two reads *r*,*s*∈*R* overlap (*r*⊓*s*), or do not overlap (*r* ⊓̸ *s*) each other. There are many studies have considered measuring the sequence overlap information between reads. One of the efficient methods is to count the number of shared *q*-mers between reads [25,26,30]. Those methods base on a feature that most *q*-mers are not shared by different genomes when *q* is sufficiently large [25,26]. For example, according to an observation conducted by this work on 100 pairs of bacterial genomes, the average ratio of common *q*-mers between the genomes is less than 1.02*%* when *q*≥30. (The details of the observation are given in Additional file 2). The feature leads to a fact that most of *q*-mer repeats in a metagenomic dataset are caused by overlaps of reads. Thus, there is a great probability that the reads sharing *q*-mers with each other (with a sufficient value of *q*) are overlapping reads.

In the proposed algorithm, a similar idea is applied to determine whether two reads *r*,*s*∈*R* overlap each other or not. Given $m, q \in \mathbb {N}$, if *r* and *s* share at least *mq*-mers, they are regarded as overlapping reads (*r*⊓*s*). Otherwise, *r* ⊓̸ *s*. The values of *m* and *q* will be discussed later in the following sections.

### Algorithms

To perform classification process, an unweighted graph *H*=(*V*,*E*) is firstly built, where *V* is a set of nodes modeling the set *R* of metagenomic reads, and *E* is a set of edges. Given $m, q \in \mathbb {N}$, ∀*r*,*s*∈*V*, each edge (*r*,*s*) represents the relation *r*⊓*s* as defined above. For a group of nodes, denoted by *G*_*i*_, we call *NS*(*G*_*i*_)=*G*_*i*_∖*S*(*G*_*i*_). We have *G*_*i*_={*S*(*G*_*i*_),*NS*(*G*_*i*_)} and *G*_*i*_⊆*V*. It is interesting that a seed *S*(*G*_*i*_) is equivalent to an *independent set* or *stable set* of a graph in which there is no pair of adjacent vertices [31]. The following describes algorithmic aspects of the proposed method in details.

#### Phase 1 - Grouping nodes and building seeds of groups

The pseudocode for this phase is provided in Algorithm 1. The node grouping in this phase is equivalent to the graph partitioning problem which can be solved by many methods [32]. In this work, a constructive method based on a greedy heuristic is suggested. Let *V*_*temp*_=*V*. Firstly, an empty group *G*_*i*_,*i*≥1 is created. Then, a node *v*∈*V*_*temp*_ is randomly chosen, removed from *V*_*temp*_ and assigned into *G*_*i*_. We denote by Neighbor(*G*_*i*_) a set of nodes *x*∈*V*_*temp*_ such that ∃*w*∈*G*_*i*_,(*w*,*x*)∈*E*. Next, other nodes *u*, where *u*∈ Neighbor(*G*_*i*_), are iteratively chosen, removed from *V*_*temp*_, and assigned into this group.

The seed building is done simultaneously with the building of groups. A greedy algorithm is applied to build seeds of the groups. Initially, the first node *v*∈*V*_*temp*_ assigned to group *G*_*i*_ is also stored in *S*(*G*_*i*_). After that, a node *u*∈*V*_*temp*_ assigned to *G*_*i*_ is only stored in its seed *S*(*G*_*i*_) if *u* is not adjacent to any of *S*(*G*_*i*_). Otherwise, *u* will be stored in *NS*(*G*_*i*_). Finally, when all groups are built, feature vectors $\phantom {\dot {i}\!}\mathbf {f}^{S(G_{i})}, \forall i \in \{1, \ldots,p\}$ will be calculated.

Sequencing errors and the existing of shared *l*-mers between genomes (even with an extremely small ratio) may lead to grouping errors. To reduce probability of the errors, the size of created groups is limited by a threshold *S*_*max*_. The process of building group *G*_*i*_ will be stopped when the size of seed *S*(*G*_*i*_), denoted by |*S*(*G*_*i*_)|, exceeds the given threshold *S*_*max*_. Note that $|S(G_{i})|=\sum _{r \in S(G_{i})} |r|$.



#### Phase 2 - Merging groups

In this phase, a *k*-means clustering algorithm [33] is used to merge the node groups *G*_*i*_,*i*∈{1,…,*p*}, created in the first phase, into clusters using feature vectors $\phantom {\dot {i}\!}\mathbf {f}^{S(G_{i})}$. Let *C*_1_,*C*_2_,…,*C*_*k*_ be a set of output clusters, and note that, *C*_*j*_⊆{*G*_1_,…*G*_*p*_}. The objective of the algorithm in this phase is to minimize the within-cluster sum of squares as the following formulation.

(2) $$  \text{minimize} \sum_{j=1}^{k} \sum_{G_{i} \in C_{j}} ||\mathbf{f}^{S(G_{i})} - \bar{\mathbf{f}}_{C_{j}}||^{2}  $$

In which $\bar {\mathbf {f}}_{C_{j}}$ is the mean of cluster *C*_*j*_, computed as follows.

(3) $$  \bar{\mathbf{f}}_{C_{j}} = \frac{\sum_{G_{w} \in C_{j}} \mathbf{f}^{S(G_{w})}}{|C_{j}|}  $$

In which |*C*_*j*_| is the number of groups in cluster *C*_*j*_.

This phase is presented by pseudocode in Algorithm 2. Firstly, the means of clusters $\bar {\mathbf {f}}_{C_{j}}^{new}\phantom {\dot {i}\!}$ are randomly chosen from feature vectors $\mathbf {f}^{S(G_{i})}\phantom {\dot {i}\!}$. Then, two following steps are repeated: (*Assignment step*) compute the distances between each $\phantom {\dot {i}\!}\mathbf {f}^{S(G_{i})}$ and the means of clusters $\bar {\mathbf {f}}_{C_{j}}^{new}\phantom {\dot {i}\!}$, and assign *G*_*i*_ to the cluster of the nearest mean *C*_*z*_ ; (*Update step*) store the current means into $\phantom {\dot {i}\!}\bar {\mathbf {f}}_{C_{j}}^{old}$ and recompute the means of recreated clusters $\phantom {\dot {i}\!}\bar {\mathbf {f}}_{C_{j}}^{new}$. The iteration stops when the algorithm converges (there is no change on mean of clusters) or a predefined number of iterations is exceeded.



### Performance evaluation

Three commonly used performance metrics, namely, *precision*, *recall*, and *F-measure* are used to evaluate the binning algorithm. Let *m* be the number of species in a metagenomic dataset, and *k* be the number of clusters returned by the binning algorithm. Let *A*_*ij*_ be the number of reads from species *j* assigned to cluster *i*. The *precision* and *recall* are defined as follows (same as used in [26]).

$$precision=\frac{\sum^{k}_{i=1}\max_{j} A_{ij}}{\sum^{k}_{i=1}\sum^{m}_{j=1} A_{ij}} $$

$$recall=\frac{\sum^{m}_{j=1}\max_{i} A_{ij}}{\sum^{k}_{i=1}\sum^{m}_{j=1} A_{ij} + \text{{\(\#\) unassigned reads}}} $$

In which *recall* presents the ratio of reads from the same species that are assigned in the same cluster, *precision* shows the ratio of reads assigned in a cluster that belong to the same species. The two metrics need to be considered together because each of them itself does not reflect the performance of a binning approach. Besides, we also use *F-measure* which emphasizes comprehensively on both *precision* and *recall*. It is defined as in [34]:

$$F-measure=2/(1/precision+ 1/recall) $$

## Experiments results and discussions

The performance of BiMeta is evaluated on simulated and real datasets. In these experiments, the number of species in data samples is assumed to be known. BiMeta is compared with several state-of-the-art binning algorithms for short or long reads. For short reads, our method is compared with MetaCluster 5.0 [26], and AbundanceBin [23] (version 1.01, February 2013). MetaCluster 3.0 [21] and MetaCluster 2.0 [20] are two recent methods for binning of long reads. Because MetaCluster 3.0 does not support fixing the number of species in datasets, for a fair comparison, in these experiments, we only compare BiMeta with MetaCluster 2.0. The computer used for the experiments is an Intel Xeon with 20GB RAM running at 2.3 GHz.

As mentioned above, when *q*≥30, most *q*-mers are not shared by genomes. Thus, *q*=30 is chosen. In addition, the precision of the first phase for read grouping and seed building of BiMeta depends on the detection of correct overlaps between reads. Using a larger value of threshold *m* (i.e., the number of shared *q*-mers between reads) can increase the probability of finding correct overlaps as well as increase the precision of this phase of the proposed algorithm. However, there is no guarantee for the algorithm to achieve better overall performance by this. Considering the classification performance on tested cases (presented in section of Parameter evaluation), we choose *m*=5 for short reads datasets, and *m*=45 for long reads datasets for the following experiments. Besides, it is realized from the observation above that groups with a length of 9000 bp are suitable for extracting genomic signatures, we set this value for the threshold *S*_*max*_.

### Datasets

#### Simulated datasets

Due to the lack of standard metagenomic datasets, simulated datasets are widely used to evaluate the performance of binning algorithms. A tool used for generating metagenomic reads is MetaSim [35] which allows us to select a sequencing model and control considered parameters (e.g., read length, genome coverage, error rate). We simulate metagenomic datasets based on the bacterial genomes which are downloaded from the NCBI (National Center for Biotechnology Information) database.

There are 25 synthetic datasets used in our experiments. Among them, 9 long reads datasets are generated as described in [27,36]. The datasets contain Roche 454 single-end long reads with the length of approximately 700 bp and sequencing error rate of 1*%*, (denoted by from R1 to R9, presented in Table 1). Besides, 16 datasets of paired-end short reads (length of approximately 80 bp) are created following the Illumina error profile with an error rate of 1*%* (denoted by from S1 to S10, and L1 to L6, presented in Table 2). A list of species or strains used to generate the datasets are given in Additional file 3.

**Table 1:** **Simulated datasets of long reads as described in [** 27 **,** 36 **]**

**Samples**	**No. of**	**Phylogenetic**	**Ratio**	**No. of**
**species**		**distance**	**reads**	
R1	2	Species	1:1	82960
R2	2	Genus	1:1	77293
R3	2	Genus	1:1	93267
R4	2	Family	1:1	34457
R5	2	Family	1:1	40043
R6	2	Order	1:1	70550
R7	3	Family and	1:1:8	290473
		Order		
R8	3	Order and	1:1:8	374830
		Phylum		
R9	6	Species, Order,	1:1:1:1:2:14	588258
		Family, Phylum,		
		and Kingdom

**Table 2:** **Simulated datasets of short reads**

**Samples**	**No. of**	**Phylogenetic**	**Ratio**	**No. of**
**species**		**distance**	**reads**	
S1	2	Species	1:1	96367
S2	2	Species	1:1	195339
S3	2	Order	1:1	338725
S4	2	Phylum	1:1	375302
S5	3	Species and	1:1:1	325400
		Family		
S6	3	Phylum and	3:2:1	713388
		Kingdom		
S7	5	Order, Order	1:1:1:4:4	1653550
		Genus, Order		
S8	5	Genus, Order	3:5:7:9:11	456224
		Order, Order		
S9	15	various distances	1:1:1:1:1:	2234168
			2:2:2:2:2:	
			3:3:3:3:3	
S10	30	various distances	4:4:4:4:4:	4990632
			6:6:6:6:6:	
			7:7:7:7:7:	
			8:8:8:8:8:	
			9:9:9:9:9:	
			10:10:10:10:10	
L1	2	Class	1:1	176688
L2	2	Class	1:2	259568
L3	2	Class	1:3	342448
L4	2	Class	1:4	425328
L5	2	Class	1:5	508209
L6	2	Class	1:6	591089

#### Real dataset

Our method is also evaluated on a real dataset obtained from the acid mine drainage (AMD) [4]. The dataset is downloaded from NCBI trace archive. It consists of 124805 Sanger reads, which are shown to belong to five dominant species: *Leptospirillum sp. Group III*, *Ferroplasma acidarmanus Type I*, *Thermoplasmatales archaeon Gpl*, *Ferroplasma sp. Type II*, and *Leptospirillum sp. Group II* with a ratio of 1:1:1:5:5, respectively. We also download scaffolds of the five species assembled from the AMD dataset for result evaluation.

### Results on simulated data

#### Results on short reads data

The performance of BiMeta are firstly compared with MetaCluster 5.0 and AbundanceBin on short read datasets with different numbers of species and different phylogenetic distances. Table 3 presents the overall *F-measure* values of the algorithms for samples from S1 to S10. BiMeta can achieve higher accuracy than both MetaCluster 5.0 and AbundanceBin in most of the cases (8 of 10 cases). When the number of species in data increases, the performance of the three algorithms decreases. Despite of this, as we can see the results on samples S9 and S10, which contain a large number of species, BiMeta still gets better *F-measure* than that of MetaCluster 5.0 and AbundanceBin.

**Table 3:** **The F-measures of MetaCluster 5.0, AbundanceBin and BiMeta on samples from S1 to S10**

**Samples**	**MetaCluster 5.0**	**AbundanceBin**	**BiMeta**
S1	67.11%	-	**98.02%**
S2	**88.68%**	72.63%	60.14%
S3	71.98%	83.53%	**97.72%**
S4	77.20%	-	**99.35%**
S5	80.08%	56.38%	**89.32%**
S6	88.74%	64.24%	**99.29%**
S7	**91.04%**	58.49%	77.24%
S8	57.94%	47.87%	**70.27%**
S9	67.56%	27.92%	**77.01%**
S10	52.17%	4.95%	**65.37%**

The symbol “-” indicates that the approaches fail to classify reads on the samples.BiMeta achieves higher F-measure in comparison with that of MetaCluster 5.0 and AbundanceBin for 8 out of 10 samples, while MetaCluster 5.0 gets the highest value for sample S2 and S7 in comparison with that of the remaining approaches.

**Figure 3:**
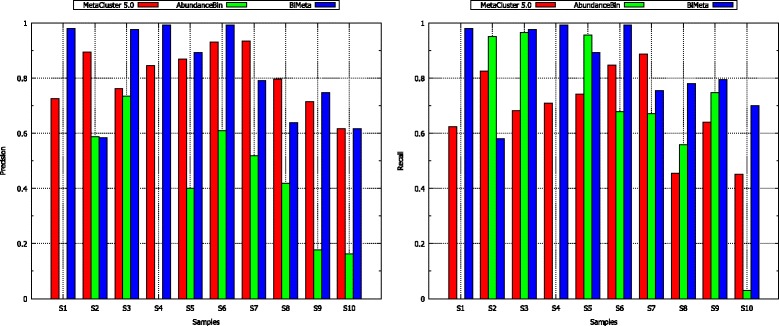
**The performance of MetaCluster 5.0, AbundanceBin and BiMeta on samples from S1 to S10.** The left bar graph shows precision values, and the right bar graph shows recall values of the algorithms. Note that AbundanceBin fails to classify reads for sample S1 and S4.

In addition, we also consider the *precision* and *recall* of the algorithms on those samples. Figure 3 demonstrates that for most of the cases the proposed method gets much higher both *recall* and *precision* values in comparison with those of MetaCluster 5.0 and AbundanceBin. Note that MetaCluster 5.0 makes an effort to get high *precision* by using the techniques of removing extremely low-coverage reads from classification process and generating more clusters if needed. However, BiMeta still gets considerably higher *precision* values than that of MetaCluster 5.0 for 6 of 10 cases.

Finding a cause for the low classification performance on sample S2 of the proposed algorithm, we randomly pick 10 pairs of non-overlapping read groups from genomes of species which are used in the sample. In each pair, a group is generated from *Lactobacillus salivarius* genome, and the other is from *Lactobacillus sanfranciscensis* genome (the two species are in the same genus). The Euclidean distance between their feature vectors is computed. From the test, we realize that the average distance computed for all pairs is very small (≈7.8×10^−4^) and much smaller than the average distance between groups in genus level (≈1.4×10^−3^) which is computed in the above observation. It is even approximately equal to the average distance between groups generated from the same species (≈7.7×10^−4^). Obviously, in this case the *l*-mer frequency distribution is not good for discriminating the two species, and this may explain the reason why our algorithm gets low performance on the sample.

**Figure 4:**
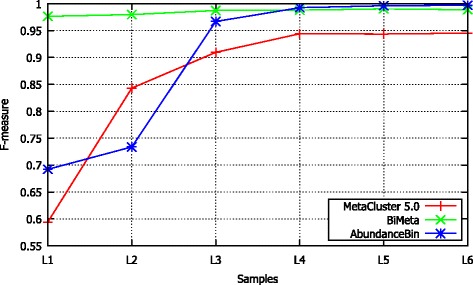
**The performance of MetaCluster 5.0, BiMeta and AbundanceBin on samples from L1 to L6.**

The abundance of species is one of the major factors affecting to the classification performance of existing binning methods. To assess the effect of this factor on BiMeta, we run the algorithm on samples from L1 to L6 and compare with MetaCluster 5.0 and AbundanceBin. The samples are generated from genomes of two species (*Eubacterium eligens* and *Lactobacillus amylovorus*), but they have different abundance ratios. Figure 4 illustrates the *F-measure* value of the three algorithms on those samples. The results demonstrate that BiMeta is stable for different ratios of species abundances, and returns better overall results comparing with the other algorithms. For more details, the proposed algorithm can achieve *F-measure* of greater than 97.5%, which means it is 4% - 38% higher than that of MetaCluster 5.0 for all of the tests. In addition, BiMeta outperforms AbudanceBin (has higher *F-measure* from 2% to 28%) when they are tested on the datasets with low abundance ratios (1:1, 1:2, and 1:3, in samples L1, L2, and L3, respectively), and it still achieves as high scores as AbundanceBin (≥98.79*%*) for the datasets with high abundance ratios (1:4, 1:5, and 1:6, in samples L4, L5 and L6, respectively). Moreover, on computational performance, the proposed algorithm needs smaller computing time than that of both AbundanceBin and MetaCluster 5.0 to execute on those samples (data is given in Additional file 3).

#### Results on long reads data

**Figure 5:**
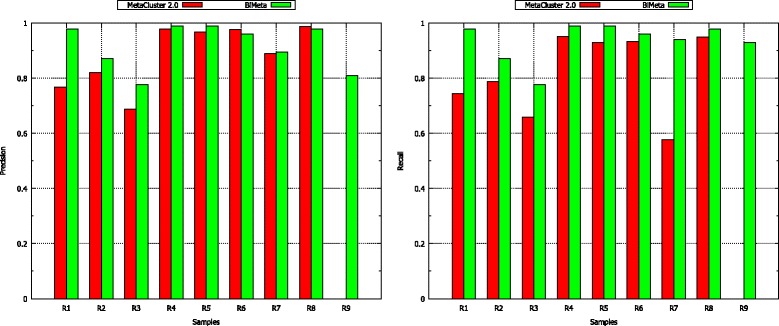
**The performance of MetaCluster 2.0 and BiMeta on samples from R1 to R9.** The left bar graph shows precision values, and the right bar graph shows recall values of the two algorithms. Note that MetaCluster 2.0 fails to perform on sample R9.

BiMeta and MetaCluster 2.0 are tested on the long read datasets from R1 to R9 (presented in Table 1). Table 4 shows that BiMeta has significantly higher *F-measure* than MetaCluster 2.0 for the all tests. With sample R9, while the proposed method achieves a high result, MetaCluster 2.0 cannot execute successfully because the number of reads are too large. Furthermore, BiMeta can obtain 0.5% - 20% higher *precision* in 6 of the 8 comparable cases, and 3% - 36% higher *recall* in those cases than MetaCluster 2.0 (Figure 5). In the tests on R7, R8, and R9, although the samples contain reads from genomes of very different abundance levels, BiMeta still reaches high accuracy (*F-measure* is from 86.42% to 97.92%).

**Table 4:** **The F-measures of MetaCluster 2.0 and BiMeta on samples from R1 to R9**

**Samples**	**MetaCluster 2.0**	**BiMeta**
R1	75.61%	**97.82%**
R2	80.40%	**87.19%**
R3	66.83%	**77.59%**
R4	96.42%	**98.94%**
R5	94.75%	**98.97%**
R6	95.40%	**96.09%**
R7	69.96%	**91.63%**
R8	96.74%	**97.92%**
R9	-	**86.42%**

The symbol “-” indicates that MetaCluster 2.0 fails to perform on sample R9. BiMeta achieves higher F-measure than that of MetaCluster 2.0 for the all samples.

### Results on real data

BiMeta and MetaCluster 2.0 are tested on the AMD dataset. To evaluate results of the two methods, BLAST tool is used to map reads of each output cluster against assembled scaffolds of the five dominant species in this dataset with BLAST E-value of ≤1*e*^−50^ (other parameters are set default). Note that from our experiments, only 69*%* percent of all reads in the dataset can be mapped to assembled scaffolds of the five species by BLAST. The numbers of BLAST hits give us a rough estimation of the classification accuracy. Although MetaCluster 2.0 gets slightly higher *precision* score than BiMeta (57.15% and 55.8%, respectively), BiMeta returns much better *recall* than MetaCluster 2.0 (88.09% and 70.93%, respectively). In total, the overall *F-measure* score of the classification achieved by BiMeta is higher than MetaCluster 2.0 (68.32% and 63.30%, respectively).

### Parameter evaluation

**Figure 6:**
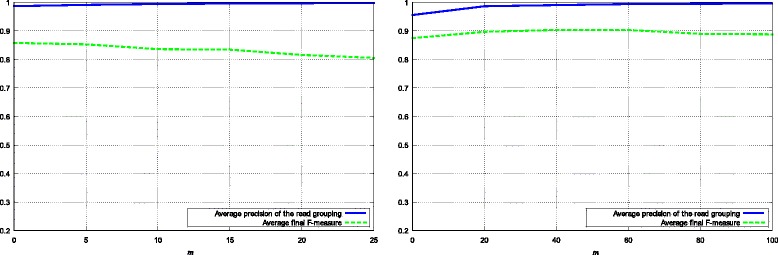
**The average** ***precision*** **of the reads grouping in phase 1, and average final** ***F-measure*** **of BiMeta with different values of** ***m*** **- the minimum number of shared** ***q*** **-mers between reads.** The left line graph shows tests results on samples from S1 to S5 (short reads). The right line graph shows tests results on samples from R1 to R5 (long reads).

In the proposed algorithm, parameter *m* is a threshold to determine whether two reads are overlapped each other or not. We conduct experiments on samples from S1 to S5 and from R1 to R5 to compute the average *precision* of the read merging in phase 1 and the average final *F-measure* of the algorithm with different values of *m*. Two graphs in Figure 6 show the effect of *m* to the performance of BiMeta. From the graphs, the proposed algorithm achieves the best results when *m* is from 0 to 5 for short read datasets, and from 20 to 65 for long reads datasets.

Besides, there is a slightly increase in the precision of the task of read grouping with respect to a decrease of *m*. For example, on datasets of short reads (samples from S1 to S5), the average *precision* is 98.68% with *m*=0, while the score is 99.82% with *m*=25. This is obviously understood because when the number of shared *l*-mers between reads is set to be larger, the probability of identified correct overlap of reads is higher.

However, as seen from the graphs, the performance of BiMeta is not proportional to the performance of this grouping task. For instance, on datasets of long reads (samples from R1 to R5), when *m* increases from 60 to 100, although the *precision* of the reads grouping increases from 99.6% to 99.82%, the final *F-measure* of BiMeta decreases from 83.43% to 80.54%. Considering the results, we realize that when *m* is larger, phase 1 of the proposed algorithm usually produces the larger number of read groups. This means that the size of the groups as well as their seeds are smaller. As a result, although the precision of the merging task is higher, because there is less information for extracting genomic signature (4-mers frequencies) from the seeds, the classification performance may decreases.

## Conclusions

This paper presents a two-phase algorithm for the binning of metagenomic reads without using reference genomes. Instead of directly clustering reads, the main idea of the proposed algorithm is to provide an additional preprocessing phase in which reads potentially belonging to the same cluster are grouped and each group is presented by a so-called seed of non-overlapping reads. The idea is motivated by a careful observation of the *l*-mer frequency distributions on sets of non-overlapping reads extracted from microbial genomes. The proposed algorithm demonstrates to be able to achieve higher performance than the state-of-the-art binning algorithms on both simulated and real metagenomic datasets. Another strength of our method is that it can work well with both short and long reads. Besides, because the second phase only performs on reads in the seed of a group, instead of the group, the algorithm runs fast with a moderate memory usage.

## Additional files

Additional file 1
**This file contains the details of datasets and results for the observation of** ***l*** **-mer frequency distribution on groups of non-overlapping reads.**

Additional file 2
**This file contains the details of datasets and results for the observation of shared** ***q*** **-mers between microbial genomes.**

Additional file 3
**This file contains the details of the datasets used in Experimental result and discussions section, and execution time of BiMeta, AbundanceBin and MetaCluster 5.0 on samples from L1 to L6.**
