# Supplementary material for: A two-phase binning algorithm using l-mer frequency on groups of non-overlapping reads
Source: Algorithms Mol Biol. 2015 Jan 16;10:2. doi: 10.1186/s13015-014-0030-4 (PMC4304631; doi:10.1186/s13015-014-0030-4)
Supplement: Additional file 1 — This file contains the details of datasets and results for the observation of l -mer frequency distribution on groups of non-overlapping reads. [file 13015_2014_30_MOESM1_ESM.pdf]

Supplementary Materials 1

The details of datasets and results for the observation of *l*-mer frequency distribution on groups of non-overlapping reads.

Table of Contents

1. Details of the tests on pairs of genomes at Genus level
2. Details of the tests on pairs of genomes at Familly level
3. Details of the tests on pairs of genomes at Species level

1. Details of the tests on pairs of genomes at Genus level

Pairs	Species/Strains	Euclidean distance
1	Arthrobacter aurescens TC1	0.00129205
	Aromatoleum aromaticum EbN1	
2	Acidithiobacillus caldus SM-1	0.000969298
	Acidithiobacillus ferrivorans SS3	
3	Acinetobacter baumannii TCDC-AB0715	0.000881397
	Acinetobacter calcoaceticus PHEA-2	
4	Acinetobacter baumannii TCDC-AB0715	0.000592282
	Acinetobacter oleivorans DR1	
5	Acinetobacter oleivorans DR1	0.000651503
	Acinetobacter calcoaceticus PHEA-2	
6	Actinobacillus pleuropneumoniae serovar 3 str. JL03	0.000545567
	Actinobacillus succinogenes 130Z	
7	Clostridium tetani E88	0.00137322
	Clostridium thermocellum ATCC 27405	
8	Corynebacterium aurimucosum ATCC 700975	0.00132225
	Corynebacterium diphtheriae 241	
9	Clostridium acetobutylicum ATCC 824	0.00190094
	Clostridium beijerinckii NCIMB 8052	
10	Clostridium beijerinckii NCIMB 8052	0.00237113
	Clostridium botulinum A str. ATCC 19397	
11	Clostridium botulinum H04402 065	0.000733144
	Clostridium cellulolyticum H10	
12	Clostridium clariflavum DSM 19732	0.00331292
	Clostridium difficile 2007855	
13	Clostridium difficile 2007855	0.000468002

	Clostridium kluyveri DSM 555	
14	Clostridium kluyveri DSM 555	0.000512852
	Clostridium lentocellum DSM 5427	
15	Ehrlichia canis str. Jake	0.000222824
	Ehrlichia chaffeensis str. Arkansas	
16	Ehrlichia chaffeensis str. Arkansas	0.00144952
	Ehrlichia ruminantium str. Gardel	
17	Enterobacter aerogenes KCTC 2190	0.000846188
	Enterobacter asburiae LF7a	
18	Enterobacter cloacae EcWSU1	0.0006097
	Enterobacter asburiae LF7a	
19	Leuconostoc kimchii IMSNU 11154	0.00137863
	Leuconostoc mesenteroides subsp. mesenteroides ATCC 8293	
20	Lactobacillus amylovorus GRL 1112	0.00130382
	Lactobacillus brevis ATCC 367	
21	Ferrimonas balearica DSM 9799	0.00520908
	Ferroglobus placidus DSM 10642	
22	Fervidicoccus fontis Kam940	0.00723474
	Ferrimonas balearica DSM 9799	
23	Fervidobacterium nodosum Rt17-B1	0.000971573
	Fervidobacterium pennivorans DSM 9078	
24	Frankia alni ACN14a	0.000624341
	Frankia symbiont of Datisca glomerata	
25	Flavobacterium branchiophilum FL-15	0.0025518
	Flavobacterium columnare ATCC 49512	
26	Flavobacterium columnare ATCC 49512	0.000794538
	Flavobacterium indicum GPTSA100-9	
27	Flavobacterium psychrophilum JIP02/86	0.000579774
	Flavobacterium indicum GPTSA100-9	
28	Francisella cf. novicida Fx1	0.00122829
	Francisella noatunensis subsp. orientalis str. Toba 04	
29	Francisella noatunensis subsp. orientalis str. Toba 04	0.000463824
	Francisella philomiragia subsp. philomiragia ATCC 25017	
30	Francisella philomiragia subsp. philomiragia ATCC 25017	0.000382518
	Francisella cf. novicida Fx1	
31	Gallibacterium anatis UMN179	0.00510855
	Gallionella capsiferriformans ES-2	

32	Geobacillus kaustophilus HTA426	0.000392288
	Geobacillus sp. C56-T3	
33	Geobacillus sp. C56-T3	0.000328194
	Geobacillus thermodenitrificans NG80-2	
34	Geobacillus thermodenitrificans NG80-2	0.000816751
	Geobacillus thermoglucosidasius C56-YS93	
35	Geobacillus thermoglucosidasius C56-YS93	0.00184899
	Geobacillus thermoleovorans CCB_US3_UF5	
36	Geobacter lovleyi SZ	0.00580897
	Geobacter metallireducens GS-15	
37	Geobacter metallireducens GS-15	0.00213207
	Geobacter sp. FRC-32	
38	Geobacter sp. FRC-32	0.00307431
	Geobacter sulfurreducens KN400	
39	Haemophilus influenzae Rd KW20	0.000582075
	Haemophilus parainfluenzae T3T1	
40	Haemophilus parainfluenzae T3T1	0.000464351
	Haemophilus parasuis SH0165	
41	Rhodobacter capsulatus SB 1003	0.00100538
	Rhodobacter sphaeroides 2.4.1	
42	Rickettsia heilongjiangensis 054	0.000362156
	Rickettsia massiliae MTU5	
43	Rickettsia peacockii str. Rustic	0.00116132
	Rickettsia prowazekii Rp22	
44	Roseiflexus castenholzii DSM 13941	0.000437446
	Roseiflexus sp. RS-1	
45	Roseobacter denitrificans OCh 114	0.000435788
	Roseobacter litoralis Och 149	
46	Ruegeria pomeroyi DSS-3	0.000974478
	Ruegeria sp. TM1040	
47	Salinispora arenicola CNS-205	0.000597411
	Salinispora tropica CNB-440	
48	Shewanella amazonensis SB2B	0.00118549
	Shewanella baltica BA175	
49	Sinorhizobium medicae WSM419	0.000465572
	Sinorhizobium meliloti 1021	
50	Streptococcus suis ST3	0.000983104
	Streptococcus thermophilus ND03	
**Average distance**		**0.001418848**

2. Details of the tests on pairs of genomes at Familly level

Pairs	Species/Strains	Euclidean distance
1	Acidiphilium cryptum JF-5	0.00116041
	Azospirillum lipoferum 4B	
2	Acidiphilium cryptum JF-5	0.00128279
	Azospirillum sp. B510	
3	Acidiphilium cryptum JF-5	0.000960068
	Rhodospirillum centenum SW	
4	Acidiphilium cryptum JF-5	0.0056813
	Rhodospirillum photometricum DSM 122	
5	Acidiphilium cryptum JF-5	0.000900461
	Rhodospirillum rubrum F11	
6	Acidiphilium multivorum AIU301	0.00119024
	Azospirillum lipoferum 4B	
7	Acidiphilium multivorum AIU301	0.00130401
	Azospirillum sp. B510	
8	Acidiphilium multivorum AIU301	0.000981188
	Rhodospirillum centenum SW	
9	Acidiphilium multivorum AIU301	0.00566501
	Rhodospirillum photometricum DSM 122	
10	Acidiphilium multivorum AIU301	0.000905204
	Rhodospirillum rubrum F11	
11	Gluconobacter oxydans 621H	0.00272485
	Magnetospirillum magneticum AMB-1	
12	Gluconobacter oxydans 621H	0.00244248
	Azospirillum lipoferum 4B	
13	Gluconobacter oxydans 621H	0.00428762
	Azospirillum sp. B510	
14	Gluconobacter oxydans 621H	0.00195362
	Rhodospirillum centenum SW	
15	Gluconobacter oxydans 621H	0.00187092
	Rhodospirillum photometricum DSM 122	
16	Gluconacetobacter xylinus NBRC 3288	0.00160728
	Magnetospirillum magneticum AMB-1	
17	Gluconacetobacter xylinus NBRC 3288	0.00187295
	Azospirillum lipoferum 4B	
18	Gluconacetobacter xylinus NBRC 3288	0.00267678
	Azospirillum sp. B510	
19	Gluconacetobacter xylinus NBRC 3288	0.00176704

	Rhodospirillum centenum SW	
20	Gluconacetobacter xylinus NBRC 3288	0.00364943
	Rhodospirillum photometricum DSM 122	
21	Helicobacter pylori v225d	0.001695
	Campylobacter concisus 13826	
22	Helicobacter pylori v225d	0.00288986
	Campylobacter curvus 525.92	
23	Helicobacter pylori v225d	0.00233894
	Campylobacter fetus subsp. fetus 82-40	
24	Helicobacter pylori v225d	0.0029078
	Campylobacter hominis ATCC BAA-381	
25	Helicobacter pylori v225d	0.0023106
	Campylobacter jejuni RM1221	
26	Helicobacter acinonychis str. Sheeba	0.00260565
	Campylobacter concisus 13826	
27	Helicobacter acinonychis str. Sheeba	0.00512735
	Campylobacter curvus 525.92	
28	Helicobacter acinonychis str. Sheeba	0.00218291
	Campylobacter fetus subsp. fetus 82-40	
29	Helicobacter acinonychis str. Sheeba	0.00160544
	Campylobacter hominis ATCC BAA-381	
30	Helicobacter acinonychis str. Sheeba	0.00138367
	Campylobacter jejuni RM1221	
31	Mycobacterium tuberculosis CCDC5079	0.00121954
	Corynebacterium aurimucosum ATCC 700975	
32	Mycobacterium tuberculosis CCDC5079	0.0022096
	Corynebacterium diphtheriae 241	
33	Mycobacterium tuberculosis CCDC5079	0.00194272
	Corynebacterium efficiens YS-314	
34	Mycobacterium tuberculosis CCDC5079	0.00305287
	Corynebacterium glutamicum ATCC 13032	
35	Mycobacterium tuberculosis CCDC5079	0.00130545
	Corynebacterium jeikeium K411	
36	Mycobacterium ulcerans Agy99	0.00130113
	Corynebacterium aurimucosum ATCC 700975	
37	Mycobacterium ulcerans Agy99	0.00275419
	Corynebacterium diphtheriae 241	
38	Mycobacterium ulcerans Agy99	0.00188054
	Corynebacterium efficiens YS-314	
39	Mycobacterium ulcerans Agy99	0.0038878
	Corynebacterium glutamicum ATCC 13032	

40	Mycobacterium ulcerans Agy99<br>Corynebacterium jeikeium K411	0.00160739
41	Rhodococcus erythropolis PR4<br>Mycobacterium abscessus	0.00144254
42	Rhodococcus erythropolis PR4<br>Mycobacterium africanum GM041182	0.00175072
43	Rhodococcus erythropolis PR4<br>Mycobacterium avium 104	0.00245791
44	Rhodococcus erythropolis PR4<br>Mycobacterium bovis AF2122/97	0.00177895
45	Rhodococcus erythropolis PR4<br>Mycobacterium canettii CIPT 140010059	0.00178442
46	Rhodococcus jostii RHA1<br>Mycobacterium chubuense NBB4	0.000571187
47	Rhodococcus jostii RHA1<br>Clavibacter michiganensis subsp. michiganensis NCPPB 382	0.0033827
48	Rhodococcus jostii RHA1<br>Clavibacter michiganensis subsp. sepedonicus	0.00266907
49	Rhodococcus jostii RHA1<br>Microbacterium testaceum StLB037	0.00144297
50	Rhodococcus jostii RHA1<br>Mycobacterium avium 104	0.000791016
**Average distance**		**0.002183272**

3. Details of the tests on pairs of genomes at Species level

Pairs	Species/Strains	Euclide distance
1	Arthrobacter aurescens TC1	0.000402109
2	Acidithiobacillus caldus SM-1	0.000338735
3	Acinetobacter baumannii TCDC-AB0715	0.000427445
4	Acinetobacter oleivorans DR1	0.000481165
5	Acinetobacter calcoaceticus PHEA-2	0.00504832
6	Actinobacillus pleuropneumoniae serovar 3 str. JL03	0.000593606
7	Clostridium tetani E88	0.00178746

8	Corynebacterium aurimucosum ATCC 700975	0.000361758
9	Clostridium acetobutylicum ATCC 824	0.000435955
10	Clostridium beijerinckii NCIMB 8052	0.00101003
11	Clostridium botulinum H04402 065	0.00199109
12	Clostridium clariflavum DSM 19732	0.000659139
13	Clostridium difficile 2007855	0.00209873
14	Clostridium kluyveri DSM 555	0.000273998
15	Ehrlichia canis str. Jake	0.000517377
16	Ehrlichia chaffeensis str. Arkansas	0.000276004
17	Enterobacter aerogenes KCTC 2190	0.000913663
18	Enterobacter cloacae EcWSU1	0.000610972
19	Leuconostoc kimchii IMSNU 11154	0.000384086
20	Lactobacillus amylovorus GRL 1112	0.00159008
21	Ferrimonas balearica DSM 9799	0.000662443
22	Fervidicoccus fontis Kam940	0.000835274
23	Fervidobacterium nodosum Rt17-B1	0.000359662
24	Frankia alni ACN14a	0.000511451
25	Flavobacterium branchiophilum FL-15	0.000794795
26	Flavobacterium columnare ATCC 49512	0.00115145
27	Flavobacterium psychrophilum JIP02/86	0.00162949
28	Francisella cf. novicida Fx1	0.00122074
29	Francisella noatunensis subsp. orientalis str. Toba 04	0.000642839
30	Francisella philomiragia subsp. philomiragia ATCC 25017	0.000303487
31	Gallibacterium anatis UMN179	0.000345561
32	Geobacillus kaustophilus HTA426	0.000390449
33	Geobacillus sp. C56-T3	0.000305441
34	Geobacillus thermodenitrificans NG80-2	0.000368147
35	Geobacillus thermoglucosidasius C56-YS93	0.000243957
36	Geobacter lovleyi SZ	0.000368481
37	Geobacter metallireducens GS-15	0.000602051
38	Geobacter sp. FRC-32	0.000710776
39	Haemophilus influenzae Rd KW20	0.000343967
40	Haemophilus parainfluenzae T3T1	0.0004172
41	Rhodobacter capsulatus SB 1003	0.00043359
42	Rickettsia heilongjiangensis 054	0.00044147
43	Rickettsia peacockii str. Rustic	0.000313373
44	Roseiflexus castenholzii DSM 13941	0.0015777
45	Roseobacter denitrificans OCh 114	0.000430132
46	Ruegeria pomeroyi DSS-3	0.000256555

47	Salinispora arenicola CNS-205	0.000435634
48	Shewanella amazonensis SB2B	0.000405258
49	Sinorhizobium medicae WSM419	0.000490665
50	Streptococcus suis ST3	0.0014154
	**Average distance**	**0.000772183**
