# Supplementary material for: A two-phase binning algorithm using l-mer frequency on groups of non-overlapping reads
Source: Algorithms Mol Biol. 2015 Jan 16;10:2. doi: 10.1186/s13015-014-0030-4 (PMC4304631; doi:10.1186/s13015-014-0030-4)
Supplement: Additional file 2 — This file contains the details of datasets and results for the observation of shared q -mers between microbial genomes. [file 13015_2014_30_MOESM2_ESM.pdf]

Supplementary Materials 2

The details of datasets and results for the observation of shared *q*-mers between microbial genomes.

We conducted a test to observe the ratio of common *q*-mers in bacterial genomes. The dataset consists of 100 pairs of genomes from different genetic distances. The ratio of common *q*-mers in genomes of each pair is computed for different values of *q* as follows.

The ratio of common *q*-mers = $nc/(nd_1 + nd_2 - nc)$.

In which, *nc* be the number of common *q*-mers in two genomes, nd_1 be the number of distinct *q*-mers in the first genome, and nd_2 be the number of distinct *q*-mers in the second genome. The figure below presents the average ratio of common *q*-mers with different values of *q*.

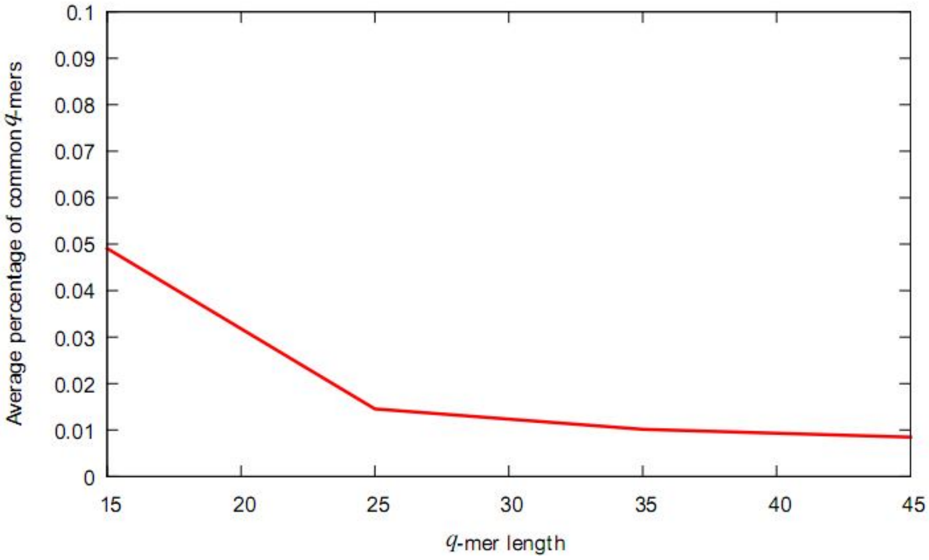

The details of datasets and results of this experiment

Pairs	Species/Strains	Ratio of common *l*-mers			
		***l*=15**	***l*=25**	***l*=35**	***l*=45**
1	Acidiphilium cryptum JF-5<br>Azospirillum lipoferum 4B	0.053591	0.000826	0.000256	0.000145
2	Acidiphilium cryptum JF-5	0.057112	0.00087	0.00027	0.000168

	Azospirillum sp. B510				
3	Acidiphilium cryptum JF-5<br>Rhodospirillum centenum SW	0.061575	0.000777	0.000231	0.000119
4	Acidiphilium cryptum JF-5<br>Rhodospirillum photometricum DSM 122	0.033701	0.000324	9.71E-05	5.78E-05
5	Acidiphilium cryptum JF-5<br>Rhodospirillum rubrum F11	0.049182	0.000429	0.000107	6.03E-05
6	Acidiphilium multivorum AIU301<br>Azospirillum lipoferum 4B	0.05344	0.000792	0.000242	0.000141
7	Acidiphilium multivorum AIU301<br>Azospirillum sp. B510	0.057072	0.000831	0.000254	0.000158
8	Acidiphilium multivorum AIU301<br>Rhodospirillum centenum SW	0.062018	0.000773	0.000227	0.000118
9	Acidiphilium multivorum AIU301<br>Rhodospirillum photometricum DSM 122	0.033991	0.000318	8.96E-05	5.33E-05
10	Acidiphilium multivorum AIU301<br>Rhodospirillum rubrum F11	0.049638	0.000428	0.000106	5.78E-05
11	Gluconobacter oxydans 621H<br>Magnetospirillum magneticum AMB-1	0.020886	0.000252	0.000104	7.24E-05
12	Gluconobacter oxydans 621H<br>Azospirillum lipoferum 4B	0.021329	0.0004	0.000168	0.000112
13	Gluconobacter oxydans 621H<br>Azospirillum sp. B510	0.021703	0.00035	0.000157	0.000106
14	Gluconobacter oxydans 621H<br>Rhodospirillum centenum SW	0.025319	0.000301	0.00012	8.15E-05
15	Gluconobacter oxydans 621H<br>Rhodospirillum photometricum DSM 122	0.015492	0.000218	9.66E-05	5.68E-05
16	Gluconacetobacter xylinus NBRC 3288<br>Magnetospirillum magneticum AMB-1	0.024037	0.000274	0.000107	7.04E-05
17	Gluconacetobacter xylinus NBRC 3288<br>Azospirillum lipoferum 4B	0.022314	0.000349	0.000154	0.000103
18	Gluconacetobacter xylinus NBRC 3288<br>Azospirillum sp. B510	0.022499	0.000322	0.000138	9.74E-05
19	Gluconacetobacter xylinus NBRC 3288<br>Rhodospirillum centenum SW	0.027084	0.000278	0.000122	8.19E-05
20	Gluconacetobacter xylinus NBRC 3288<br>Rhodospirillum photometricum DSM 122	0.018091	0.000209	9.41E-05	5.78E-05
21	Helicobacter pylori v225d	0.010971	0.000308	0.000215	0.000167

	Campylobacter concisus 13826				
22	Helicobacter pylori v225d<br>Campylobacter curvus 525.92	0.008837	0.000305	0.000218	0.000174
23	Helicobacter pylori v225d<br>Campylobacter fetus subsp. fetus 82-40	0.011021	0.000336	0.000231	0.00019
24	Helicobacter pylori v225d<br>Campylobacter hominis ATCC BAA-381	0.014205	0.000305	0.00021	0.000166
25	Helicobacter pylori v225d<br>Campylobacter jejuni RM1221	0.017842	0.000373	0.00024	0.000187
26	Helicobacter acinonychis str. Sheeba<br>Campylobacter concisus 13826	0.011105	0.000311	0.000214	0.000165
27	Helicobacter acinonychis str. Sheeba<br>Campylobacter curvus 525.92	0.00879	0.000316	0.00022	0.000175
28	Helicobacter acinonychis str. Sheeba<br>Campylobacter fetus subsp. fetus 82-40	0.011383	0.000323	0.000218	0.00018
29	Helicobacter acinonychis str. Sheeba<br>Campylobacter hominis ATCC BAA-381	0.014715	0.000301	0.000201	0.000158
30	Helicobacter acinonychis str. Sheeba<br>Campylobacter jejuni RM1221	0.01857	0.000376	0.000232	0.000186
31	Mycobacterium tuberculosis CCDC5079<br>Corynebacterium aurimucosum ATCC 700975	0.016841	0.000246	0.000123	8.37E-05
32	Mycobacterium tuberculosis CCDC5079<br>Corynebacterium diphtheriae 241	0.008249	0.000233	0.000145	0.000109
33	Mycobacterium tuberculosis CCDC5079<br>Corynebacterium efficiens YS-314	0.023746	0.00032	0.000142	9.26E-05
34	Mycobacterium tuberculosis CCDC5079<br>Corynebacterium glutamicum ATCC 13032	0.010387	0.00022	0.000125	9.01E-05
35	Mycobacterium tuberculosis CCDC5079<br>Corynebacterium jeikeium K411	0.017647	0.000345	0.000156	0.00011
36	Mycobacterium ulcerans Agy99<br>Corynebacterium aurimucosum ATCC 700975	0.018194	0.000239	0.000104	7.26E-05
37	Mycobacterium ulcerans Agy99<br>Corynebacterium diphtheriae 241	0.008613	0.000212	0.000124	9.64E-05
38	Mycobacterium ulcerans Agy99<br>Corynebacterium efficiens YS-314	0.026133	0.000297	0.000123	8.20E-05
39	Mycobacterium ulcerans Agy99<br>Corynebacterium glutamicum ATCC 13032	0.011284	0.0002	0.000109	7.93E-05
40	Mycobacterium ulcerans Agy99	0.018614	0.000316	0.000136	9.47E-05

	Corynebacterium jeikeium K411				
41	Rhodococcus erythropolis PR4<br>Mycobacterium abscessus	0.032166	0.000654	0.000233	0.000142
42	Rhodococcus erythropolis PR4<br>Mycobacterium africanum GM041182	0.03059	0.000448	0.00016	0.000104
43	Rhodococcus erythropolis PR4<br>Mycobacterium avium 104	0.041997	0.000632	0.000201	0.000116
44	Rhodococcus erythropolis PR4<br>Mycobacterium bovis AF2122/97	0.030533	0.000448	0.00016	0.000104
45	Rhodococcus erythropolis PR4<br>Mycobacterium canettii CIPT 140010059	0.030733	0.000442	0.000157	0.000103
46	Rhodococcus jostii RHA1<br>Mycobacterium chubuense NBB4	0.074817	0.001206	0.000359	0.000205
47	Rhodococcus jostii RHA1<br>Clavibacter michiganensis subsp. michiganensis NCPPB 382	0.063935	0.000391	0.00011	6.37E-05
48	Rhodococcus jostii RHA1<br>Clavibacter michiganensis subsp. sepedonicus	0.062386	0.000382	0.000113	6.06E-05
49	Rhodococcus jostii RHA1<br>Microbacterium testaceum StLB037	0.064757	0.000372	0.000102	5.82E-05
50	Rhodococcus jostii RHA1<br>Mycobacterium avium 104	0.072491	0.001038	0.000296	0.000157
51	Arthrobacter aurescens TC1<br>Aromatoleum aromaticum EbN1	0.020229	3.41E-05	8.42E-06	4.04E-06
52	Acidithiobacillus caldus SM-1<br>Acidithiobacillus ferrivorans SS3	0.014139	0.001115	0.00061	0.00046
53	Acinetobacter baumannii TCDC-AB0715<br>Acinetobacter calcoaceticus PHEA-2	0.131116	0.063645	0.040039	0.031063
54	Acinetobacter baumannii TCDC-AB0715<br>Acinetobacter oleivorans DR1	0.109767	0.04701	0.02748	0.020587
55	Acinetobacter oleivorans DR1<br>Acinetobacter calcoaceticus PHEA-2	0.195388	0.104095	0.067349	0.053325
56	Actinobacillus pleuropneumoniae serovar 3 str. JL03<br>Actinobacillus succinogenes 130Z	0.017256	0.002073	0.001075	0.000782
57	Clostridium tetani E88<br>Clostridium thermocellum ATCC 27405	0.019823	0.00012	5.34E-05	2.81E-05

58	Corynebacterium aurimucosum ATCC 700975<br>Corynebacterium diphtheriae 241	0.010625	0.001267	0.000691	0.000492
59	Clostridium acetobutylicum ATCC 824<br>Clostridium beijerinckii NCIMB 8052	0.048906	0.000492	0.000162	9.77E-05
60	Clostridium beijerinckii NCIMB 8052<br>Clostridium botulinum A str. ATCC 19397	0.058419	0.000481	0.000164	9.81E-05
61	Clostridium botulinum H04402 065<br>Clostridium cellulolyticum H10	0.025429	9.62E-05	3.41E-05	1.23E-05
62	Clostridium clariflavum DSM 19732<br>Clostridium difficile 2007855	0.031506	7.69E-05	3.49E-05	2.09E-05
63	Clostridium difficile 2007855<br>Clostridium kluyveri DSM 555	0.04432	0.000107	3.02E-05	1.53E-05
64	Clostridium kluyveri DSM 555<br>Clostridium lentocellum DSM 5427	0.027807	7.20E-05	2.29E-05	8.88E-06
65	Ehrlichia canis str. Jake<br>Ehrlichia chaffeensis str. Arkansas	0.052207	0.011644	0.004454	0.002793
66	Ehrlichia chaffeensis str. Arkansas<br>Ehrlichia ruminantium str. Gardel	0.032025	0.00378	0.001544	0.001131
67	Enterobacter aerogenes KCTC 2190<br>Enterobacter asburiae LF7a	0.041591	0.008314	0.004209	0.002998
68	Enterobacter cloacae EcWSU1<br>Enterobacter asburiae LF7a	0.090464	0.0354	0.021166	0.0163
69	Leuconostoc kimchii IMSNU 11154<br>Leuconostoc mesenteroides subsp. mesenteroides ATCC 8293	0.027991	0.010982	0.008471	0.007466
70	Lactobacillus amylovorus GRL 1112<br>Lactobacillus brevis ATCC 367	0.00601	0.000326	0.000176	0.000114
71	Ferrimonas balearica DSM 9799<br>Ferroglobus placidus DSM 10642	0.002619	6.25E-07	0	0
72	Fervidicoccus fontis Kam940<br>Ferrimonas balearica DSM 9799	0.001235	1.81E-06	0	0
73	Fervidobacterium nodosum Rt17-B1<br>Fervidobacterium pennivorans DSM 9078	0.022471	0.008456	0.007644	0.007329
74	Frankia alni ACN14a<br>Frankia symbiont of Datisca glomerata	0.099534	0.002669	0.000972	0.000586
75	Flavobacterium branchiophilum FL-15<br>Flavobacterium columnare ATCC 49512	8.34E-05	0	0	0
76	Flavobacterium columnare ATCC 49512	0.037122	0.001369	0.000592	0.000403

	Flavobacterium indicum GPTSA100-9				
77	Flavobacterium psychrophilum JIP02/86<br>Flavobacterium indicum GPTSA100-9	0.033162	0.000947	0.000371	0.000251
78	Francisella cf. novicida Fx1<br>Francisella noatunensis subsp. orientalis str. Toba 04	0.049798	0.012356	0.005969	0.004085
79	Francisella noatunensis subsp. orientalis str. Toba 04<br>Francisella philomiragia subsp. philomiragia ATCC 25017	0.23284	0.130136	0.083074	0.06448
80	Francisella philomiragia subsp. philomiragia ATCC 25017<br>Francisella cf. novicida Fx1	0.060274	0.021076	0.013198	0.010466
81	Gallibacterium anatis UMN179<br>Gallionella capsiferriformans ES-2	0.005335	6.91E-05	2.72E-05	1.49E-05
82	Geobacillus kaustophilus HTA426<br>Geobacillus sp. C56-T3	0.460798	0.374116	0.318441	0.29044
83	Geobacillus sp. C56-T3<br>Geobacillus thermodenitrificans NG80-2	0.053992	0.013658	0.006336	0.00451
84	Geobacillus thermodenitrificans NG80-2<br>Geobacillus thermoglucosidasius C56-YS93	0.023635	0.004094	0.002642	0.002211
85	Geobacillus thermoglucosidasius C56-YS93<br>Geobacillus thermoleovorans CCB_US3_UF5	0.023909	0.00392	0.002439	0.002019
86	Geobacter lovleyi SZ<br>Geobacter metallireducens GS-15	0.014598	0.000679	0.000303	0.000217
87	Geobacter metallireducens GS-15<br>Geobacter sp. FRC-32	0.017985	0.00117	0.000451	0.000283
88	Geobacter sp. FRC-32<br>Geobacter sulfurreducens KN400	0.016685	0.00083	0.000341	0.000227
89	Haemophilus influenzae Rd KW20<br>Haemophilus parainfluenzae T3T1	0.034913	0.00966	0.005387	0.003994
90	Haemophilus parainfluenzae T3T1<br>Haemophilus parasuis SH0165	0.016895	0.002411	0.00124	0.000908
91	Rhodobacter capsulatus SB 1003<br>Rhodobacter sphaeroides 2.4.1	0.06446	0.003134	0.000955	0.000492
92	Rickettsia heilongjiangensis 054<br>Rickettsia massiliae MTU5	0.523623	0.382843	0.29407	0.253271
93	Rickettsia peacockii str. Rustic	0.089304	0.028678	0.012143	0.007651

	Rickettsia prowazekii Rp22				
94	Roseiflexus castenholzii DSM 13941<br>Roseiflexus sp. RS-1	0.056443	0.005	0.001435	0.000773
95	Roseobacter denitrificans OCh 114<br>Roseobacter litoralis Och 149	0.1204	0.053303	0.03071	0.022734
96	Ruegeria pomeroyi DSS-3<br>Ruegeria sp. TM1040	0.032634	0.002204	0.000799	0.00051
97	Salinispora arenicola CNS-205<br>Salinispora tropica CNB-440	0.144206	0.037764	0.018762	0.013013
98	Shewanella amazonensis SB2B<br>Shewanella baltica BA175	0.014119	0.001281	0.000608	0.000433
99	Sinorhizobium medicae WSM419<br>Sinorhizobium meliloti 1021	0.113863	0.037104	0.017663	0.011952
100	Streptococcus suis ST3<br>Streptococcus thermophilus ND03	0.012741	0.002262	0.001327	0.000989
**Average**		0.049063	0.014535	0.010142	0.008477
