# Supplementary material for: A two-phase binning algorithm using l-mer frequency on groups of non-overlapping reads
Source: Algorithms Mol Biol. 2015 Jan 16;10:2. doi: 10.1186/s13015-014-0030-4 (PMC4304631; doi:10.1186/s13015-014-0030-4)
Supplement: Additional file 3 — This file contains the details of the datasets used in Experimental result and discussions section, and execution time of BiMeta, AbundanceBin and MetaCluster 5.0 on samples from L1 to L6. [file 13015_2014_30_MOESM3_ESM.pdf]

Supplementary Materials 3

Table of Contents

1. Details of long read datasets
2. Details of short read datasets
3. Execution time of BiMeta, AbundanceBin and MetaCluster 5.0 on samples from L1 to L6.

1. Details of long read datasets

ID	Species/Strain	Coverage
R1	Bacillus halodurans C-125	7
	Bacillus subtilis BSn5	7
R2	Gluconobacter oxydans 621H	7
	Granulibacter bethesdensis CGDNIH1	7
R3	Escherichia coli LF82	7
	Yersinia pestis Z176003	7
R4	Methanocaldococcus jannaschii DSM 2661	7
	Methanococcus maripaludis C5	7
R5	Pyrobaculum aerophilum str. IM2	7
	Thermofilum pendens Hrk 5	7
R6	Gluconobacter oxydans 621H	7
	Rhodospirillum rubrum F11	7
R7	Gluconobacter oxydans 621H	5
	Granulibacter bethesdensis CGDNIH1	5
	Nitrobacter hamburgensis X14	40
R8	Escherichia coli UM146	5
	Pseudomonas putida BIRD-1	5
	Bacillus anthracis str. H9401	40
R9	Escherichia coli HS	5
	Pseudomonas putida F1	5
	Thermofilum pendens Hrk 5	5
	Pyrobaculum aerophilum str. IM2	5
	Bacillus anthracis str. H9401	10
	Bacillus subtilis BSn5	70

2. Details of short read datasets

ID	Species/Strain	Coverage
S1	Mycoplasma suis KI3806	10
	Mycoplasma putrefaciens KS1	10
S2	Lactobacillus salivarius UCC118	10
	Lactobacillus sanfranciscensis TMW 1.1304	10
S3	Lactobacillus salivarius UCC118	10
	Bacillus selenitireducens MLS10	10
S4	Borrelia burgdorferi JD1	10
	Escherichia coli APEC O1	10
S5	Lactobacillus salivarius UCC118	10
	Lactobacillus sanfranciscensis TMW 1.1304	10
	Aerococcus urinae ACS-120-V-Col10a	10
S6	Borrelia burgdorferi JD1	30
	Thermofilum pendens Hrk 5	20
	Escherichia coli APEC O1	10
S7	Actinobacillus pleuropneumoniae serovar 5b str. L20	10
	Aliivibrio salmonicida LFI1238	10
	Haemophilus somnus 129PT	10
	Pasteurella multocida 36950	40
	Vibrio cholerae M66-2	40
S8	Haemophilus somnus 129PT	3
	Pasteurella multocida 36950	5
	Actinobacillus pleuropneumoniae serovar 5b str. L20	7
	Vibrio cholerae M66-2	9
	Aliivibrio salmonicida LFI1238	11
S9	Azospirillum sp. B510	5
	Bacillus amyloliquefaciens LL3	5
	Bartonella clarridgeiae 73	5
	Bifidobacterium animalis subsp. lactis B420	5
	Bordetella avium 197N	5
	Caldicellulosiruptor lactoaceticus 6A	10
	Desulfovibrio vulgaris DP4	10
	Ehrlichia canis str. Jake	10
	Geobacter sulfurreducens PCA	10
	Haemophilus somnus 2336	10
	Helicobacter cetorum MIT 00-7128	15

	Lactobacillus amylovorus GRL1118	15
	Mesotoga prima MesG1.Ag.4.2	15
	Rickettsia akari str. Hartford	15
	Streptococcus thermophilus JIM 8232	15
S10	Acidaminococcus intestini RyC-MR95	4
	Acinetobacter baumannii ATCC 17978	4
	Advenella kashmirensis WT001	4
	Alicycliphilus denitrificans K601	4
	Anaeromyxobacter sp. Fw109-5	4
	Bacillus amyloliquefaciens Y2	6
	Bacteroides fragilis NCTC 9343	6
	Bifidobacterium animalis subsp. lactis AD011	6
	Borrelia afzelii Pko	6
	Borrelia turicatae 91E135	6
	Burkholderia ambifaria AMMD	7
	Caldicellulosiruptor owensensis OL	7
	Candidatus Amoebophilus asiaticus 5a2	7
	Chelativorans sp. BNC1	7
	Clostridium cellulolyticum H10	7
	Coxiella burnetii RSA 331	8
	Dechlorosoma suillum PS	8
	Desulfitobacterium hafniense Y51	8
	Edwardsiella ictaluri 93-146	8
	Erwinia amylovora ATCC 49946	8
	Escherichia coli DH1	9
	Ferrimonas balearica DSM 9799	9
	Finegoldia magna ATCC 29328	9
	Fluviicola taffensis DSM 16823	9
	Frankia sp. CcI3	9
	Gallibacterium anatis UMN179	10
	Geobacillus sp. C56-T3	10
	Geobacter sp. FRC-32	10
	Gordonia polyisoprenivorans VH2	10
	Haemophilus ducreyi 35000HP	10
L1	Eubacterium eligens ATCC 27750	7
	Lactobacillus amylovorus GRL1118	7
L2	Eubacterium eligens ATCC 27750	7
	Lactobacillus amylovorus GRL1118	14
L3	Eubacterium eligens ATCC 27750	7
	Lactobacillus amylovorus GRL1118	21
L4	Eubacterium eligens ATCC 27750	7

	Lactobacillus amylovorus GRL1118	28
L5	Eubacterium eligens ATCC 27750	7
	Lactobacillus amylovorus GRL1118	35
L6	Eubacterium eligens ATCC 27750	7
	Lactobacillus amylovorus GRL1118	42

3. **Execution time of BiMeta, AbundanceBin and MetaCluster 5.0 on samples from L1 to L6.**

**Samples**	**MC 5.0** (Second)	**AbundanceBin** (Second)	**BiMeta** (Second)
L1	502.41	266	**198.44**
L2	508.57	504	**254.35**
L3	555.7	436	**338.14**
L4	572.24	516	**424.98**
L5	607.55	652	**510.02**
L6	645.85	724	**631.53**
